# Clonal haematopoiesis and cardiovascular disease, defining risk, filling gaps, and framing the future

**DOI:** 10.1038/s44325-026-00127-4

**Published:** 2026-05-21

**Authors:** Alec P. Morley, Paul Carter, Rupen Hargreaves, Dorina-Gabriela Condurache, Michael Anderson, Sean Wen, Zahra Raisi-Estabragh

**Affiliations:** 1 Gonville and Caius College, University of Cambridge, Cambridge, UK; 2 Cambridge University Hospitals NHS Foundation Trust, Cambridge, UK; 3 Victor Phillip Dahdaleh Heart and Lung Research Institute, University of Cambridge, Cambridge, UK; 4 MRC Weatherall Institute of Molecular Medicine, University of Oxford, Oxford, UK; 5 William Harvey Research Institute, NIHR Barts Biomedical Research Centre, Barts Heart Centre, Barts Health NHS Trust, Queen Mary University of London, London, UK; 6 Health Organisation, Policy, Economics, Centre for Primary Care & Health Services Research, University of Manchester, Manchester, UK; 7 Department of Health Policy, London School of Economics and Political Science, London, UK; 8 Centre for Genomics Research, Discovery Sciences, BioPharmaceuticals R&D, AstraZeneca, Cambridge, UK; 9 Department of Haematology, Wellcome-MRC Cambridge Stem Cell Institute, Jeffrey Cheah Biomedical Centre, University of Cambridge, Cambridge, UK

**Keywords:** Cardiology, Diseases, Genetics, Stem cells

## Abstract

Clonal haematopoiesis (CH) refers to the clonal expansion of haematopoietic stem cells in the absence of overt haematological malignancy and is common in older individuals. Recent evidence has heralded CH as a novel determinant of cardiovascular disease. This review explores the associations of CH with ischaemic heart disease, heart failure, atrial fibrillation and cardio-oncology, highlighting biases in the literature, exploring effects of mutation and clone size, concluding with clinical implications.

## Introduction

Haematopoiesis begins with multipotent haematopoietic stem cells (HSCs), which can self-renew and differentiate into more specialised blood cells. Somatic mutations may give proliferative advantage to HSCs, leading to their clonal expansion. Established risk factors for these mutations are older age^1^, history of cytotoxic exposures such as chemoradiotherapy^2–4^, tobacco smoking^5^, sex^6^, obesity^7^, telomere length^8^, genetic polymorphisms^6,9^ (such as the *TCL1A* locus), ancestry^10^ and medications^11^. More than 70 different gene drivers have been implicated in clonal expansion of HSCs^12^, conferring different fitness effects^13^, with, for example, splicing factor and *JAK2* mutations having relatively higher fitness compared to *DNMT3A* mutations, which have relatively lower fitness^14^. However, rates of clonal expansion can vary across the lifetime^15^, and be influenced by germline genetics^14^ and environmental exposures^16^, and are not driven by mutation alone^15^. The proportion of mutant cells found in the blood, termed the variant allele fraction (VAF), can be measured, which reflects clone size at that point, with the increase over time corresponding to clonal fitness.

While people with clonal expansion of HSCs have an increased relative risk of malignant transformation, this requires additional mutations, and most never develop malignancies^17^, unless driven by higher fitness mutations (such as within splicing factors). Clonal expansion of HSCs with demonstrable preleukemic driver mutations to a VAF > 2% in the absence of malignancy has historically been termed clonal haematopoiesis of indeterminate potential (CHIP), although more recently updated to clonal haematopoiesis (CH) given increased understanding of prognostically significant disease associations and of smaller clones not meeting the 2% VAF threshold. Another form of clonal expansion is characterised by chromosomal abnormalities and is termed mosaic chromosomal alteration (mCA). mCA encompasses three subtypes, namely autosomal mCA, mosaic loss of chrX (mLOX) and mosaic loss of chrY (mLOY).

**Fig. 1:**
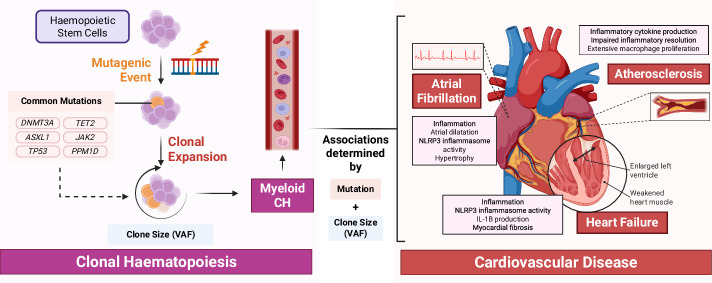
An overview of the pathogenesis of CH. CH clonal haematopoiesis, VAF variant allele fraction. Figure was created in BioRender. Morley, A. (2026) https://BioRender.com/qxzcp2l.

Population studies demonstrate that CH is common, especially in older adults, affecting more than 10% of people over 65-years old^1^. CH is linked to a heightened risk of cardiovascular^6,18–37^, oncological^6,38^, metabolic^7,39,40^, hepatic^41^, respiratory^42,43^ and renal^44^ conditions as well as a steep 40% increase in mortality risk^17^, which has been supported by a recent meta-analysis^45^. Among these, cardiovascular diseases (CVDs) are the most important contributor to excess mortality^18^ – with growing evidence indicating associations with increased risk of atherosclerotic disease^6,18–23^, heart failure^6,20,23–29^ and atrial fibrillation^6,30–33^, as shown in Fig. 1.

While some researchers have heralded CH as a novel CVD determinant, others highlight the risk of confounding with major known vascular risk factors, such as age and smoking. The bidirectional relationship between CH and CVD is recognised, and reverse causation has been raised as a potential factor in augmenting apparent associations of CH with adverse cardiovascular outcomes^20,46^. The issue of survival bias is also notable across many studies, with the omission of individuals at the highest mortality risk in existing cohorts potentially distorting some observed relationships with age-related illnesses. Methodological strategies to disentangle cause-and-effect and mitigate key biases are important for understanding the independent causal relationships of CH.

The existing VAF threshold for CH is set pragmatically at 2% (reflecting 4% of heterozygotic cells), based in part on technical limits of detection. While modern high-resolution sequencing technologies permit detection of driver mutations at very low VAF levels, the clinical utility of ultrasensitive measurements and optimal VAF threshold for CH is not yet established. In addition, the number of driver genes included in CH definitions and the panel of CH genes varies widely even across key publications^6,9,12,17^, and a high proportion of CH is with unknown drivers^47^.

Emerging evidence suggests differential disease risk across CH driver mutations and clone sizes. However, many of these analyses have limited statistical power for rarer mutations and smaller clones, limiting the robustness of their conclusions. Unravelling mutation and clone size-specific relationships with CVD subtypes is essential for understanding biological mechanisms and identifying novel therapeutic targets. Furthermore, there is an urgent need for a mutually agreed consensus on defining CH, given that associations currently described in the literature are dependent upon often differing CH definitions, and lead to limited cross-comparability and result in heterogeneity in reported effect sizes^20,46,48^.

This narrative review evaluates the latest research linking CH with specific cardiovascular conditions, varying by mutation and clone size, and highlights sources of bias and gaps in current literature. We further discuss clinical implications and the current position of CH in relation to the Wilson–Jungner screening criteria.

## Literature search

Electronic literature databases (MEDLINE and Embase) were searched using relevant search terms to cover all published literature from 1946 to December 2025, yielding 299 relevant publications post-screening. The number of publications relating to CH has increased substantially since 2017, from 24 published in 2017 to an almost exponential increase of over 200 in the last three years. The most relevant publications are selected for discussion in this review and grouped into three major CVD categories of atherosclerotic disease, heart failure (HF) and atrial fibrillation (AF). We also discuss CH in the context of cardio-oncology, a special population of patients at heightened risk of both CH and cardiovascular complications. The CH mutations associated with CVD discussed in this review are summarised in Table 1 and the key observational studies included in the review are summarised in Table 2.

**Table 1:** Key known mutation-CH associations which are implicated in CVDs

Gene (Protein)	Role	Mutation type	Proportion of CH
*DNMT3A* (DNA methyltransferase)	Epigenetic regulator	Loss-of-function, (non-R882/R882 missense)	~40–60%^1,2,6,9,10,12,13,15,17,20,45–47,62,63,88,101^
*TET2* (Tet dioxygenase)	Epigenetic DNA demethylation	Loss-of-function	~10–35%^1,2,6,9,10,12,13,15,17,20,45–47,62,63,88,101^
*ASXL1* (Polycomb regulator)	Chromatin regulator	Frameshift/nonsense	~3–15%^1,2,6,9,10,12,13,15,17,20,45–47,62,63,88,101^
*JAK2* (tyrosine kinase)	Myeloproliferative driver	V617F missense	~1–5%^1,2,6,9,10,12,13,17,20,45–47,62,63^
*TP53* (tumour suppressor)	DNA damage response	Missense or truncating	~1–8%^1,2,6,9,10,12,13,15,17,20,45–47,62,63,101^
*PPM1D* (p53 pathway phosphatase)	DNA damage response	Truncating mutations in exon 5 and 6	~3–8%^2,6,9,10,12,15,20,45,47,88^

*CH* clonal haematopoiesis, *CVD* cardiovascular disease.

**Table 2:** Summary of key observational studies of CH and CVD

First author, year	Study population	Sample size	Age (mean/median/range)	Ethnic background	CH definition	Disease focus	Effect size (HR/OR, 95% CI)
Atherosclerotic disease							
Jaiswal et al., 2017^18^	Adults from 4 case–control studies (BioImage, MDC, ATVB, PROMIS); no known haematologic malignancy	8255 (4726 CAD cases and 3529 controls)	60–70 years (BioImage and MDC)	Predominantly White/European (BioImage/MDC/ATVB); South Asian (PROMIS)	WES-defined CH; ≥1 somatic mutation in 74 myeloid malignancy-associated genes at VAF ≥ 2%; large clone CH: VAF ≥ 10%	CAD and early-onset MI	CH was associated with CAD (HR: 1.9, 95% CI: 1.4–2.7, *p* < 0.001) and early-onset MI (OR: 4.0, 95% CI: 2.4–6.7, *p* < 0.001); VAFs ≥10% associated with coronary artery calcification (OR: 12; 2.4–64, *p* = 0.002) in the BioImage cohort
Jaiswal et al., 2017^18^	Adults from 2 case–control studies (BioImage, MDC) and 3 prospective cohorts (JHS, FUSION, FHS); no known haematologic malignancy	4578 (240 CH cases and 4438 controls)	60–70 years (BioImage and MDC)	Predominantly White/European (BioImage/MDC/FUSION/FHS); African–American (JHS)	WES-defined CH; ≥1 somatic mutation in 74 myeloid malignancy-associated genes at VAF ≥ 2%; gene-specific analyses (*DNMT3A*, *TET2*, *ASXL1* and *JAK2*);	CAD and early-onset MI	*DNMT3A*, *ASXL1*, *JAK2* and other-CH all associated with CAD (*DNMT3A*: HR: 1.7, 95% CI: 1.1–2.6, *p* = 0.01; *ASXL1*: HR: 2.0 95% CI: 1.0–3.9, *p* = 0.05; *JAK2*: HR: 12, 95% CI: 3.8–38.4, *p* < 0.001; other: HR: 2.2, 95% CI: 1.3–3.7, *p* = 0.002), *TET2, ASXL1, JAK2* and other mutations all associated with early onset MI (*p* ≤ 0.02).
Kar et al., 2022^6^	Adults from UKB with WES	200,453	38–72 years	Predominantly White/European	WES of 43 CH genes; somatic mutations filtered against predefined CH driver variants; stratified by gene (*DNMT3A*, *TET2*, *ASXL1*, *JAK2*, *SRSF2* + *SF3B1*) and clone size (VAF < 0.1 vs. ≥0.1)	Incident CAD and MI	Overall-CH and incident CAD (HR: 1.36, 95% CI: 1.22–1.53, *p* = 2.00 × 10^−7^, with correction for smoking) Overall-CH and incident MI (HR:1.32, 95% CI: 1.23–1.42, *p* = 1.25 × 10^−12^, with correction for smoking)
Zekavat et al., 2023^19^	Adults from MGBB and UKB; leukaemia-free	50,122 (MGBB: 12,465 [657 CH cases, 11,808 controls]; UKB: 37,657 [2,194 CH cases, 35,463 controls])	MGBB mean age 60.12 years in CH cases, 46.13 years in controls; UKB 60.59 years in CH cases, 56.81 years in controls	86.1% White CH cases, 80% controls (MGBB); 100% White CH cases & controls (UKB)	WES-defined CH; somatic mutations in haematological malignancy-related genes at VAF ≥ 2%; gene-specific analyses (*DNMT3A, TET2*, *TP53*, *PPM1D*, *ASXL1* and splicing factors); large clones VAF > 10%	Incident CAD	Gene-specific associations with CAD including *TET2*-CH (HR: 2.05, 95% CI: 1.54 − 2.71, *p* = 6.3 × 10^−7^), *TP53*-CH (HR: 2.31, 95% CI: 1.03 − 5.16, p = 0.042), *PPM1D*-CH (HR: 2.63, 95% CI: 1.40 − 4.95, p = 2.7 × 10^−3^), *ASXL1*-CH (HR: 1.73, 95% CI: 1.09 − 2.76, *p* = 0.021),
Stacey et al., 2023^20^	Adults from UKB and Icelandic deCODE cohort	176,219 (UKB: 130,709; deCODE: 45,510)	Median 58.4 years (UKB); 53.0 years (deCODE)	Predominantly White/European	Mutational-barcode CH from WGS; ≥20 low-VAF singleton mosaic mutations (VAF 0.1–0.25) after artefact/germline filtering	CAD, MI, revascularisation composite, HF; all-cause mortality	No association with CVD in UKB (HR: 1.01, 95% CI 0.94–1.08, *p* = 0.88)
Heimlich et al., 2024^21^	Prospective cohort of patients undergoing invasive coronary angiography	1142	Median 68 years (CH) vs. 59 years (non-CH)	73% White	Targeted deep sequencing; ≥1 CH driver mutation at VAF ≥ 2%	Coronary stenosis (left main, LAD), including obstructive/non-obstructive lesions	Overall-CH with left main stenosis (OR: 2.44, 95% CI: 1.40–4.27, *p* = 0.021) *TET2*-CH with obstructive LAD stenosis (OR: 2.62, 95% CI: 1.34–5.11, *p* = 0.039)
Diez-Diez et al., 2024^22^	PESA healthy cohort	3692	Median 45 years	Predominantly White/European	Targeted sequencing of 54 CH-driver genes; CH = myeloid CH genes; large clones VAF ≥ 2%	Subclinical incident atherosclerosis	CH increased risk of de novo femoral atherosclerosis at 3 years (OR:1.53, 95% CI: 1.12–2.07, *p* = 0.007) with larger clones (VAF ≥ 2%) showing a higher risk (OR: 2.06, 95% CI 1.22 − 3.37, *p* = 0.005).
Zuriaga et al., 2024^23^	Adults from MGBB and UKB; leukaemia-free	474,417 (MGBB: 37,181; UKB: 437, 236)	MGBB: mean 61.7 ± 11.3 years; UKB: mean 56.5 ± 8.1 years	88.4% White (MGBB); 83.7% White (UKB)	WES-defined CH; somatic mutations in haematological malignancy-related genes at VAF ≥ 2%; gene-specific analyses (*TET2*, *DNMT3A* and *ASXL1*)	Composite CVD outcome (CAD, MI, ischaemic stroke and all-cause mortality) and MI; prevalent (MGBB); incident (UKB)	Within MGBB, associations of overall-CH, *TET2*-CH and *ASXL1*-CH with prevalent composite CVD outcome. Within UKB, associations of overall-CH, *TET2*-CH, *DNMT3A*-CH and *ASXL1*-CH with incident composite CVD, associations of overall-CH and *ASXL1*-CH with incident MI (overall-CH: HR: 1.09, 95% CI: 1.02 − 1.15, *p* = 7.5 × 10^−3^; *ASXL1*-CH: HR: 1.29, 95% CI: 1.10 − 1.51, *p* = 1.5 × 10^−3^)
**Heart Failure**							
Dorsheimer et al, 2018^24^	Chronic ischaemic HF patients taken from trials examining the effects of intracoronary administration of autologous bone marrow cells	200	Median age 65 [56–72] years	-	Deep amplicon sequencing of 56 CH/myeloid genes in bone marrow; ≥1 somatic mutation with VAF ≥ 2%; prognostic analyses focused on *DNMT3A*/*TET2*	Death or HF hospitalisation	Higher mortality with HF hospitalisation among those with *DNMT3A* or *TET2* mutations (HR 2.1, 95% CI: 1.1–4.0, *p* = 0.02). Higher mortality associated with clonal size (VAF)
Pascual-Figal et al., 2021^25^	Single-centre cohort of ambulatory patients with ischaemic and non-ischaemic HF with reduced left ventricular ejection fraction	62	Mean 74 ± 7 years	-	Targeted deep sequencing (54 CH genes); ≥1 driver mutation with VAF ≥ 2%	HF death or HF hospitalisation	*DNMT3A* or *TET2* mutations showed accelerated HF progression in terms of death (HR: 2.79, 95% CI 1.31 − 5.92, *p* = 0.008), death or HF hospitalisation (HR: 3.84, 95% CI: 1.84 − 8.04, *p* < 0.001) and HF-related death or HF hospitalisation (HR: 4.41, 95% CI: 2.15 − 9.03, *p* < 0.001)
Kar et al., 2022^6^	Adults from UKB with WES	200,453	38–72 years	Predominantly White/European	WES of 43 CH genes; somatic mutations filtered against predefined CH driver variants; stratified by gene (*DNMT3A*, *TET2*, *ASXL1*, *JAK2*, *SRSF2* + *SF3B1*) and clone size (VAF < 0.1 vs. ≥0.1)	Incident HF	Overall-CH and incident HF (HR: 1.21, 95% CI: 1.07–1.36, p = 1.38 × 10^−2^) Large clone CH and incident HF (HR: 1.30, 95% CI: 1.09 − 1.55, *p* = 1.76 × 10^−2^)
Stacey et al., 2023^20^	Adults from UKB and Icelandic deCODE cohort	176,219 (UKB 130,709; deCODE 45,510)	Median 58.4 years (UKB); 53.0 years (deCODE)	Predominantly White/European	Mutational-barcode CH from WGS; ≥20 low-VAF singleton mosaic mutations (VAF 0.1–0.25) after artefact/germline filtering	CAD, MI, revascularisation composite, HF; all-cause mortality	No association with CVD in UKB (HR: 1.01, 95% CI 0.94–1.08, *p* = 0.88)
Schuermans et al., 2024^28^	Adults from JHS and WHI; no prevalent HF	8090 (JHS 2927; WHI 5163)	JHS: median 56 years [46–65]; WHI: median 67 years [62–72]	100% Black (JHS); 66% non-Hispanic White (WHI)	WGS-defined CH; ≥1 somatic mutation in 74 leukaemia-associated genes at VAF > 2%. Gene-specific subtypes: *DNMT3A* and *TET2*. Large clone CH: VAF > 10%	HFpEF and HFrEF	*TET2*-CH was significantly associated with HFpEF (HR: 2.35, 95% CI: 1.34 − 4.11, *p* = 0.003)
Sikking et al., 2024^29^	Non-ischaemic DCM patients from the Maastricht Cardiomyopathy Registry	520	Median 57 years [49–65]	Predominantly White/European	CH driver mutations with VAF ≥ 0.01% detected using the single-molecule molecular inversion probe technique	Non-ischaemic DCM prognosis	CH with a VAF cutoff of 0.36% was associated with a higher risk of cardiac death (HR: 2.33, 1.24 − 4.40). CH with a VAF cutoff of 0.06% was associated with a high risk of all-cause mortality (HR: 1.72, 95% CI 1.10 − 2.69)
Zuriaga et al., 2024^23^	Adults from MGBB and UKB; leukaemia-free	474,417 (MGBB: 37,181; UKB: 437, 236)	MGBB: mean 61.7 ± 11.3 years; UKB: mean 56.5 ± 8.1 years	88.4% White (MGBB); 83.7% White (UKB)	WES-defined CH; somatic mutations in haematological malignancy-related genes at VAF ≥ 2%; gene-specific analyses (*TET2*, *DNMT3A* and *ASXL1*)	Composite CVD outcome (CAD, MI, ischaemic stroke, and all-cause mortality) and HF; prevalent (MGBB); incident (UKB)	Within MGBB, associations of overall-CH, *TET2*-CH and *ASXL1*-CH with prevalent composite CVD outcome, association of overall-CH with prevalent HF (OR: 1.08, 95% CI 1.00 − 1.16, *p* = 0.05) Within UKB, associations of overall-CH, *TET2*-CH, *DNMT3A*-CH and *ASXL1*-CH with incident composite CVD, associations of overall-CH, *DNMT3A*-CH and *ASXL1*-CH with incident HF (overall-CH: HR: 1.22, 95% CI: 1.15 − 1.30, *p* = 7.3 × 10^−11^; *DNMT3A*-CH; HR: 1.15, 95% CI: 1.05 − 1.24, *p* = 1.2 × 10^−3^; *ASXL1*-CH: HR: 1.40, 95% CI: 1.19 − 1.64, *p* = 4.8 × 10^−5^)
**Atrial Fibrillation**							
Kar et al., 2022^6^	Adults from UKB with WES	200,453	38–72 years	Predominantly White/European	WES of 43 CH genes; somatic mutations filtered against predefined CH driver variants; stratified by gene (*DNMT3A*, *TET2*, *ASXL1*, *JAK2*, *SRSF2* + *SF3B1*) and clone size (VAF < 0.1 vs. ≥0.1)	Prevalent AF	Overall-CH and AF (HR: 1.13, 95% CI: 1.04–1.22, *p* = 1.68 × 10^−2^, with correction for age and smoking)
Ahn et al., 2024^30^	Adults with non-valvular AF and non-AF healthy controls from Seoul National University Hospital; prognosis evaluated in AF patients in UKB	25,631 (Korean discovery cohort: 4345; UKB: 21,286)	50–79 years	East Asian (Korean discovery cohort); predominantly White (UKB)	Somatic mutation in any of 24 CH-associated genes with VAF ≥ 1.5%, detected by deep-targeted sequencing	AF	Higher prevalence of overall-CH, *DNMT3A*-CH and *TET2*-CH in AF within discovery cohort (overall-CH: HR: 1.44, 95% CI: 1.16 − 1.77, *p* < 0.001; *DNMT3A*-CH; HR: 1.57, 95% CI: 1.21 − 2.04, *p* < 0.001; *TET2*-CH: HR: 1.72, 95% CI: 1.16 − 2.55, *p* = 0.008). CH in AF associated with higher incidence of composite HF, ischaemic stroke or death (HR:1.32; 1.20-1.45, *p* < 0.001).
Schuermans et al., 2024^31^	Adults from UKB without prevalent arrhythmias/ haematologic malignancy	410,702	Median 58 years [50–63]	Predominantly White/European	WES-defined CH; VAF ≥ 2% (any CH) and VAF ≥ 10% (large clone CH); gene subtypes (*DNMT3A*, *TET2*, *ASXL1*, *JAK2*, *PPM1D*, *TP53* and spliceosome)	Incident arrhythmias: brady, supra/ventricular, AF, cardiac arrest; myocardial fibrosis in the imaging subset	Any CH: HR 1.09 to 1.16 across arrhythmia subtypes (including AF: HR: 1.11, 95% CI: 1.04–1.18, *p* = 0.001), cardiac arrest (HR: 1.29, 95% CI: 1.06–1.58, *p* = 0.012) and myocardial fibrosis (OR: 1.19, 95% CI: 1.01 − 1.41, *p* = 0.035), with increases in magnitude of all associations for large clone CH
Saadatagah et al., 2024^32^	Adults form ARIC and UKB without haematologic malignancy/mitral valve disease	199,982 (ARIC: 4131; UKB: 195,85)	ARIC: mean 75.8 ± 5.2 years; UKB: mean 56.4 ± 8.1 years	77% White (ARIC); 94% White (UKB)	WES-defined CH; VAF ≥ 2% (any CH) and VAF ≥ 10% (large clone CH); gene subtypes (*DNMT3A*, *TET2* and *ASXL1*)	AF	Any large clone CH: incident AF (HR: 1.12, 95% CI: 1.01 − 1.25, *p* = 0.036) *TET2*-CH: incident AF (HR: 1.18, 95% CI: 1.01 − 1.38, *p* = 0.043), with risk increasing for larger clone size (HR: 1.29, 95% CI: 1.05 − 1.59, *p* = 0.015) *ASXL1*-CH: incident AF (HR: 1.33, 95% CI: 1.02 − 1.73, *p* = 0.033), with risk increasing for larger clone size (HR: 1.45, 95% CI: 1.02 − 2.07, *p* = 0.040)
Teng et al., 2025^33^	Patients with *JAK2*^V617F^–positive MPN	439	Median 57 years [12–87]	Predominantly East Asian	*JAK2*^V617F^ gene mutation; additional somatic mutation profiling by NGS (*TET2*, *DNMT3A*, *ASXL1*, *FAT1* and *EP300*)	AF	*TET2* mutation independently predicted AF (HR: 4.36, 95% CI: 1.05–18.06, *p* = 0.042); IL-1β elevation also predicted AF (HR: 5.48, 95% CI: 1.55–28.12, *p* = 0.012)

*AF* atrial fibrillation, *ARIC* Atudy, *ATVB* Atherosclerosis, Thrombosis and Vascular Biology Italian Study, *CAD* coronary artery disease, *CH* clonal haematopoiesis, *CI* confidence interval, *CVD* cardiovascular disease, *DCM* dilated cardiomyopathy, *FHS* Framingham Heart Study, FUSION Finland–United States Investigation of NIDDM [Non–Insulin-Dependent Diabetes Mellitus] Genetics, *HF* heart failure, *HFpEF* heart failure with preserved ejection fraction, *HFrEF* heart failure with reduced ejection fraction, *HR* hazard ratio, *JHS* Jackson Heart Study, *LAD* left anterior descending artery, *MDC* Malmö Diet and Cancer, *MGBB* Mass General Brigham Biobank, *MI* myocardial infarction, *NGS* next-generation sequencing, *OR* odds ratio, *PESA* Progression of Early Subclinical Atherosclerosis, *PROMIS* Pakistan Risk of Myocardial Infarction Study, *UKB* UK Biobank, *VAF* variant allele fraction, *WES* whole-exome sequencing, *WGS* whole-genome sequencing, *WHI* Women’s Health Initiative.

## Atherosclerotic disease

CH has been repeatedly linked with increased risk of atherosclerotic CVDs, independent of traditional risk factors (such as smoking, obesity and hyperlipidaemia). In a seminal work, Jaiswal et al.^18^ evaluated the relationship of CH with atherosclerotic disease across multiple cohorts, presenting a prospective and a complementary retrospective analysis, which provided insights into mutation-specific risks. Firstly, using two retrospective case-control cohorts capturing 7,245 participants, they demonstrated four times greater odds of early-onset myocardial infarction (MI) in those with CH compared to non-carriers (OR: 4.0, 95% CI: 2.4–6.7, *p* < 0.001). Second, in a nested prospective study using data from two cohorts including 1010 patients, those with CH had a nearly 2-fold greater risk of incident MI or coronary revascularisation (HR: 1.9, 95% CI: 1.4–2.7, *p* < 0.001).

Heimlich et al.^21^ investigated relationships of CH with the extent and pattern of coronary atherosclerosis in 1142 patients undergoing clinically indicated coronary angiography. Individuals with CH had a similar risk of having any coronary stenosis or obstructive stenoses overall compared with controls, but they were more likely to have prognostically adverse patterns of disease, with higher odds of obstructive disease in the left main stem (OR: 2.44, 95% CI: 1.40–4.27, *p* = 0.021). The cross-sectional nature of this analysis precludes temporal separation of CH onset from development of atherosclerotic disease, limiting causal inference, in particular due to reverse causation. While the researchers adjust for major demographic and vascular risk factors, residual confounding cannot be fully excluded. Furthermore, the mechanism through which CH would specifically target specific parts of the coronary anatomy is unclear.

Other researchers have explored the idea of reverse causation in the associations between CH and atherosclerotic CVD. Heyde et al.^49^ demonstrated that atherosclerosis increases proliferation rates of both human and mouse haematopoietic stem cells and proposed that the increased CH of varying mutation subtypes associated with atherosclerotic disease could, instead, be due to atherosclerosis uniformly expanding clones across driver genes to detectable levels, thereby increasing CH detection. Indeed, experimental atherogenesis accelerated the expansion of *Tet2*^*−/−*^ cells, although whether this occurs uniformly across other gene mutations or with heterozygotic mutations found much more commonly in CH was not studied. Carter et al.^50^ performed similar experiments, and although experimental atherogenesis also promoted faster expansion of *Tet2*^*−/−*^ cells, atherogenesis had no impact on clonal expansion of heterozygotic *Tet2* mutant cells, or in the context of other driver genes (*Dnmt3a* or *Jak2*). This questions the relevance of an expansion of homozygotic mutations and further demonstrates that any effect is unlikely to be uniform across all genes. This is supported by an analysis of 4184 otherwise healthy middle-aged individuals in the Progression of Early Subclinical Atherosclerosis (PESA) study^22^, which demonstrated a unidirectional association of CH mutations with de-novo femoral atherosclerosis over 6 years of prospective follow-up. In addition, Zuriaga et al.^23^ demonstrate associations of overall-CH with incident coronary artery disease (HR: 1.07, 95% CI: 1.02–1.13, *p* = 5.8 × 10^−3^) and incident MI (HR: 1.09, 95% CI: 1.02–1.15, *p* = 7.5 × 10^−3^) in an analysis of 437,236 UK Biobank (UKB) participants.

Jaiswal et al.^18^ conducted a meta-analysis of five prospective studies, demonstrating heightened but differential risk of coronary artery disease across common driver mutations. *DNMT3A*, *TET2* and *ASXL1* mutations were linked to between 1.7- and 2.0-times greater risk, whilst *JAK2*^V617F^ mutation was associated with 12.0 times greater risk. Given that *DNMT3A* is the most common CH driver mutation at a population level, this analysis likely represents true differences in risk rather than variations in statistical power, unlike the small cohort for *JAK2*-CH. However, Heimlich et al.^21^ report no association of *DNMT3A*-CH with the measured angiographic outcomes (any stenosis, obstructive stenosis, anatomically significant stenosis). Consistent with these observations, other analyses in large biobanks^6,9,20^ and using Mendelian Randomisation^6^ (MR) report no or weak associations of *DNMT3A*-CH with atherosclerotic outcomes. This is also somewhat corroborated in experimental studies by Rauch et al.^51^ and Carter et al.^50^, who found that bone marrow chimeric models of *Dnmt3a*^+/−^ CH did not develop augmented atherosclerosis. Rauch et al.^51^ found that homozygotic perturbations in *Dnmt3a*^−/−^ mice did accelerate atherosclerosis and posited some mechanistic insight as to why an association of *DNMT3A*-CH with CAD *may* exist^51^. An augmented pro-inflammatory cytokine profile (increased IL-1β, IL-6 and Cxcl chemokines) in mutant *Dnmt3a*^−/−^ cells was observed, which is convergent to *Tet2*^−/−^ clones and the authors propose similar processes drive coronary atherosclerosis in these two common mutations. We therefore highlight that despite some limited epidemiologic and experimental evidence of *DNMT3A*-CH augmenting atherosclerosis, this is largely unsupported by analysis of adequately powered human cohorts.

In comparison to *DNMT3A* mutations, *TET2* mutations have demonstrated stronger associations with atherosclerotic coronary artery disease. Mutation-specific analysis conducted by Jaiswal et al.^18^ demonstrated an 8-fold higher odds of early-onset MI (OR: 8.3, 95% CI: 1.2–352, *p* = 0.020) and a non-significantly increased risk of incident coronary artery disease (HR: 1.9, 95% CI: 1.0–3.7, *p* = 0.060). Both analyses are severely underpowered, including a total of 13 and 29 participants from pooled studies, respectively, with marked imprecision of the estimate for early-onset MI. A high-risk pattern of coronary artery atherosclerosis was reported by Heimlich et al.^21^ amongst a small cohort of 42 individuals with *TET2-*CH who exhibited a significantly increased risk of obstructive left anterior descending artery stenosis (OR: 2.62, 95% CI: 1.34–5.11, *p* = 0.039), whereas no associations were found for *DNMT3A*-CH. Carter et al.^50^ found augmented atherosclerosis in *Tet2* experimental models to be specific to female mice. Thus far, sex-dependent effects of CH on CVD in humans have been understudied, but this raises the possibility that CH underlies some of the late-life rise in CVD in women, which warrants further investigation.

Zuriaga et al.^23^ examined associations of *DNMT3A-, TET2-* and *ASXL1*-CH with prevalent and incident cardiovascular outcomes in the Mass General Brigham Biobank (MGBB, *n* = 37,181) and UKB (*n* = 437,236), respectively. Within cross-sectional analyses in the MGBB subset, *TET2-CH* (OR: 1.14, 95% CI: 1.00–1.31, *p* = 0.050) and *ASXL1-CH* *(*OR*:* 1.51, 95% CI: 1.23–1.86, *p* = 8.5 × 10^−5^) were significantly associated with a composite of any CVD outcome, while relationships with *DNMT3A-CH* were non-significant. Associations with prevalent disease-specific associations (coronary artery disease, MI, stroke and HF) were non-significant for all mutations. In prospective associations within UKB over more than 14 years of follow-up, all three mutations were significantly linked to a higher incidence of any cardiovascular events. *TET2-CH* showed no significant associations with any of the disease-specific incident cardiovascular outcomes, while *ASXL1*-CH was significantly linked to incident coronary artery disease and MI, and *DNMT3A*-CH was linked to a higher risk of incident HF. Augmented atherosclerosis by *TET2-*CH is supported by experimental work by Fuster et al.^52^ who examined the relationship of *Tet2* mutations in murine models with atherosclerosis and identified that *Tet2* mutations accelerate atherosclerosis in atherosclerosis-prone (*Ldlr*^−/−^) models. They further examined the mechanisms underlying this association and found evidence of increased NLRP3-mediated IL-1β secretion, thus indicating inflammatory cytokine overproduction may be responsible; furthermore, therapeutically intervening with NLRP3 inhibition provided a greater atheroprotective effect with *Tet2*-mutant *Ldlr*^−/−^ mice than non-mutants. Such interventions will need to be tailored to mutational subtypes with augmented NLRP3 function; Carter et al.^50^ found that whilst *Tet2* mutant macrophages displayed increased NLRP3 activity, the opposite was true for *Dnmt3a* mutations that had lower activity, whilst *Jak2* was unaffected. Thus, targeting NLRP3 may have a specific benefit in *TET2-*CH.

Consistent with the previously mentioned observations by Zuriaga et al.^23^, *ASXL1* mutations also conferred a higher risk of coronary artery disease in the meta-analysis by Jaiswal et al.^18^ (HR: 2.0, 95% CI: 1.0–3.9, *p* = 0.050). Sato et al.^53^ examined the association of *Asxl1* mutant macrophages in atherosclerosis-prone (*Ldlr*^*−/−*^) mice, implicating a disinhibition of the innate immune system and NF-κB activation (leading to NLRP3 inflammasome activation), thus contributing to similar inflammatory cytokine overproduction as seen with *TET2* mutations.

Myeloproliferative neoplasms driven by *JAK2* mutations increase cardiovascular risk through a number of mechanisms, including heightened risk of both arterial and venous thromboses^54–56^. It is therefore unsurprising that Jaiswal et al.^18^ observed a very large association of *JAK2*-CH with coronary artery disease (HR: 12.0, 95% CI: 3.8–38.4, *p* < 0.001). However, given the small sample size in this study, the association requires further examination. Low-level *JAK2* mutations with a prevalence of 3.1% have been observed within a Danish study^57^ of 19,958 individuals, which could suggest *JAK2* is a population-level risk factor for CVD, including atherosclerosis. Fidler et al.^58^ demonstrated increased atherosclerotic plaque size in *Jak2*^*V617F*^ murine models, and although they also implicated a pro-inflammatory phenotype including increased IL-1β production, mechanistically this diverged from *Tet2* murine models with the AIM2 inflammasome (rather than NLRP3) driving oxidative stress and DNA damage with large necrotic cores. Furthermore, work conducted by Wang et al.^59^ which transplanted 100% *Jak2* mutant cells into murine models, better modelling myeloid neoplasia than CH per se, identified increased neutrophil content in early atherosclerotic lesions, and that *Jak2*^*V617F*^ erythrocytes were more susceptible to erythrophagocytosis, which drove progression of late plaques. This highlights that *JAK2*-CH differs in the pathophysiology of atherosclerosis, both compared to other driver genes, but also temporally, necessitating further investigation.

*TP53* mutations are believed to contribute to atheromatous disease through augmenting cellular proliferation rather than the inflammatory mechanisms previously discussed. Zekavat et al.^19^ found *TP53*-CH to be associated with incident atherosclerotic disease across a variety of vascular beds in UKB and MGBB, with an over 2-fold increase in coronary artery disease (HR: 2.31, 95% CI: 1.03–5.16, *p* = 0.042) and a 5-fold increase in peripheral artery disease (HR: 4.98, 95% CI: 1.23–20.09, *p* = 0.024). Through investigation of *Trp53*^−/−^ murine models, they noted an increase in p53-deficient macrophage proliferation in aortic atherosclerotic lesions, which highlights the heterogeneity of mechanisms leading to atheromatous disease driven by disparate CH mutations. We highlight that biallelic knock-out of p53 may represent a rare and more extreme phenotype than is observed in human *TP53*-CH, although the authors report that this recapitulates the dominant negative effect of *TP53* mutations in CH.

A phenome-wide association study (PheWAS) in the UKB conducted by Lin et al.^60^ revealed that autosomal mCA, mLOX and mLOY were associated with acute MI (autosomal mCA: OR: 1.38, 95% CI: 1.26–1.51, *p* = 7.69 × 10^−12^; mLOX: OR: 1.75, 95% CI: 1.49–2.06, *p* = 1.64 × 10^−11^; mLOY: OR: 1.79, 95% CI: 1.71–1.88, *p* = 2.78 × 10^−125^). Autosomal mCA and mLOY were additionally associated with atherosclerosis (autosomal mCA: OR: 1.98, 95% CI: 1.43–2.73, *p* = 3.15 × 10^−5^; mLOY: OR: 2.37, 95% CI: 1.94–2.89, *p* = 1.36 × 10^−17^), and mLOX with chronic ischaemic heart disease (OR: 1.64, 95% CI: 1.45–1.87, *p* = 2.91 × 10^−14^). This further supported prior work by Haitjema et al.^61^ who examined associations of mLOY with a cohort of 610 men who underwent carotid endarterectomy from the Athero-Express Biobank; mLOY was associated secondary major cardiovascular events (HR: 2.28, 95% CI: 1.11–4.67, *p* = 0.02), but was not associated with atheroma size (OR: 2.15, 95% CI: 1.06–4.76, *p* = 0.04) after Bonferroni correction.

In conclusion, various driver genes have been associated with atherosclerosis, and overlapping mechanisms have been implicated, which may form a mechanistic framework. Broadly, they can be classified into: (1) inflammatory cytokine overproduction (*TET2-*, *JAK2-*, *ASXL1-*, with weaker evidence for *DNMT3A-CH*), (2) impaired inflammatory resolution (*JAK2-* and *TET2-CH*) and (3) extensive macrophage proliferation (*JAK2-* and *TP53-CH*). These classifications are not mutually exclusive, with significant crossover between mechanisms.

The associations of some CH mutational subtypes and atherosclerosis are, however, based on small numbers of participants, limiting the precision and statistical power to estimate true effects, particularly for mutations with low population prevalence. Further longitudinal studies across different cohorts are warranted to validate existing findings and provide greater confidence in causal associations of CH with atherosclerotic outcomes.

## Heart failure

Recent findings support the role of CH as a key driver and prognostic indicator for HF of various aetiologies. In an early study, Dorsheimer et al.^24^ report the prevalence of CH in 18.5% of 200 patients with chronic ischaemic HF, which is higher than that reported in unselected cohorts^17,62,63^. Furthermore, they found that *DNMT3A-* and *TET2-CH* independently doubled the risk of death or HF hospitalisation over a median prospective follow-up period of 4.4 years, highlighting CH as a marker of prognosis in this context. This risk was also present for those with small clone fractions, but those with larger clone sizes had an incrementally poorer prognosis. Sano et al.^64^ investigated the role of *DNMT3A* and *TET2* mutations in an Ang II murine model of HF, revealing associations with greater macrophage and T-cell infiltration of the myocardium and adverse cardiac remodelling (via inflammation). These initial works provided the foundation for further investigation of the role of CH within HF, whilst also indicating inflammation as a key mediator of associations with *DNMT3A-* and *TET2-CH*.

Subsequent studies have revealed inconsistencies in the associations of CH with HF, highlighting potential variations in the relationship by driver mutation and HF subtype (HF with reduced or preserved ejection fraction – HFrEF and HFpEF). Zuriaga et al.^23^ report no significant associations between prevalent HF and overall-CH or CH related to three common driver mutations (*DNMT3A*, *TET2* or *ASXL1*) in 37,181 participants from the MGBB. Conversely in their prospective analysis of 437,236 UKB participants, they report significant associations with incident HF and overall-CH (HR: 1.22, 95% CI: 1.15–1.30, *p* = 7.3 × 10^−11^), *DNMT3A-*CH (HR: 1.15, 95% CI: 1.05–1.24, *p* = 1.2 × 10^−3^), and *ASXL1*-CH (HR: 1.40, 95% CI: 1.19–1.64, *p* = 4.8 × 10^−5^). In a smaller prospective analysis of 8090 individuals from The Jackson Heart Study and Women’s Health Initiative, Scheurmans et al.^28^ did not find a significant association between *DNMT3A*-CH (or overall-CH) and incident HF (either HFpEF or HFrEF). Divergence was found for *TET2*-CH, which was associated with elevated risk of HFpEF (HR: 2.35, 95% CI: 1.34–4.11, *p* = 0.003), but not HFrEF. The concurrent increase in CRP levels found in *TET2*-CH raised the possibility of an inflammation-driven process, with the intersection between inflammation and metabolism increasingly recognised to be central to HFpEF pathogenesis. However, the lack of association with HFrEF raises the question of whether HF associations are driven by HFpEF or whether the findings by Scheurmans et al.^28^ were due to differences in the size and composition of the underlying populations studied, including a more racially diverse and female-predominant cohort. These observations may also be attributed to the biologically opposing functions of *DNMT3A* and *TET2*, with roles in gene repression and activation, respectively, and some evidence for opposing actions on inflammasome activity^50^. However, previous studies^51^ have identified a paradoxical concordance of macrophage phenotypes, which has yet to be reconciled within the context of HFpEF. Yu et al.^27^ also found no significant association with *DNMT3A*-CH and incident HF, although this contrasts with evidence from Pascual-Figal et al.^25^ who found significantly faster HFrEF progression with a greater risk of overall mortality, HF death or hospitalisation. Furthermore, a recent MR study^65^ suggested causal associations of *DNMT3A*-CH with adverse remodelling patterns of larger left and right ventricular end-diastolic volumes.

The associations of *TET2*-CH and HF have been found relatively consistently throughout the literature, with increased incidence (of HFpEF)^28^, faster progression^24^, supportive pre-clinical models^66^ and an implication of NLRP3 activation and IL-1β production leading to adverse cardiac remodelling. Similarly, less common driver mutations, including *ASXL1* and *JAK2*^27,67^ have been associated with a higher incidence of HF, with an effect graded to clonal size. Such associations were significant with or without antecedent coronary artery disease, indicating the effect of CH is not solely through driving ischaemic disease – lending supportive evidence for alternative mechanisms (such as inflammation), which perhaps also explains greater consistency in reported association with HFpEF subtypes. Finally, despite associations of CH with HF being attenuated by smaller clone size, Sikking et al.^29^ identified a 21% prevalence of CH in a study of 520 dilated cardiomyopathy patients, with clones below 2% VAF having prognostic significance of all-cause mortality (HR: 1.72, 95% CI: 1.10–2.69, *p* = 0.017), with a VAF threshold of 0.06%. This finding further strengthens the impact of CH in non-ischaemic HF and highlights that very small clones are not benign and can demonstrably influence morbidity and mortality.

The UKB PheWAS conducted by Lin et al.^60^ revealed a positive association between mLOY and HF (OR: 1.67, 95% CI: 1.51–1.85, *p* = 1.14 × 10^−22^). These findings are supported by a further prospective UKB study^68^, which demonstrated increased risk of HF death among individuals with mLOY (HR: 1.76, 95% CI: 1.01–3.05, *p* = 0.045). The mechanisms underlying such an association were explored by Sano et al.^68^ using murine models lacking chromosome Y, which demonstrated a fibrotic phenotype and decreased cardiac output, with effects mitigated by transforming growth factor β1–neutralising antibody administration.

Most current studies are limited by sample size, cross-sectional study designs, and a lack of detailed phenotyping of CH and HF required to make reliable and precise conclusions. Future studies should strive to provide more granular definitions of HF, considering underlying inherited and acquired aetiologies, as well as to distinguish preserved and reduced ejection fraction phenotypes.

## Atrial fibrillation

Atrial fibrillation (AF) is the most common sustained arrhythmia, associated with advancing age, chronic inflammation, obesity and atrial fibrosis. CH has been implicated to contribute to pro-inflammatory and fibrotic changes within the myocardium; therefore, it is unsurprising that associations have been described between CH and AF, albeit in a mutation and clone size-dependent manner^32^.

The largest prospective study to date, conducted by Saadatagah et al.^32^, assessed incident AF risk amongst 199,982 participants across the Atherosclerosis Risk in Communities (ARIC) study (median follow-up 7.0 years) and UKB (median follow-up 12.2 years). Incident AF risk was increased with any *TET2-*CH (HR: 1.18, 95% CI: 1.01–1.38, *p* = 0.043) and large clone *TET2*-CH (HR: 1.29, 95% CI: 1.05–1.59, *p* = 0.015); the importance of small clone CH was not specifically assessed. These findings are corroborated by a retrospective case-control study within an East Asian cohort from Ahn et al.^30^ who reported a 65% increased odds of AF with *TET2-*CH (OR: 1.65, 95% CI: 1.05–2.60, *p* = 0.030); *TET2*-CH was also associated with atrial enlargement and increased ventricular filling pressures – remodelling patterns predisposing to AF. Notably, though, atrial size was not significantly different in CH across a range of driver genes, including *TET2*-CH in the longitudinal study by Saadatagah et al.^32^. Given that atrial dilatation and remodelling are key factors in the pathogenesis of AF, further studies are required to elucidate the effect of CH on atrial size and function.

Experimental investigations with *Tet2*^−/−^ mice conducted by Lin et al.^69^ examined the mechanisms underlying such associations of *TET2*-CH with AF, which implicated NLRP3-inflammasome activation and calcium dysregulation; attenuation of AF risk was observed with oral administration of an NLRP3 inhibitor. This supported observational findings of increased IL-6 with large clone *TET2*-CH (which is known to be upregulated by IL-1β produced by the NLRP3-inflammasome^70^) in Saadatagah et al.’s^32^ study and provides a convincing explanation of a pro-inflammatory state mediating the association of *TET2*-CH with AF.

*ASXL1*-CH also demonstrates an association with increased AF risk; in an observational study conducted by Schuermans et al.^31^ of 410,702 UKB participants, any size of *ASXL1*-CH associated with a 1.23-fold increase of AF (HR: 1.23, 95% CI 1.05–1.45, *p* = 0.012), albeit solely in the minimally adjusted model. However, this finding was replicated in Saadatagah et al.’s^32^ analysis, which reported increased risk of AF with any *ASXL1*-CH (HR: 1.33, 95% CI: 1.02–1.73, *p* = 0.033). Individuals with large clone *ASXL1*-CH demonstrate larger indexed left ventricular mass (B-coefficient per 1-SD increase in log-transformed mass: 0.26, 95% CI: 0.12–0.40, *p* = 3.5 × 10^−4^) and greater high-sensitivity troponin T (B-coefficient: 0.21, 95% CI: 0.10–0.32, *p* = 1.8 × 10^−4^), indicative of both hypertrophy and cardiomyocyte injury. Although these changes in cardiac structure and function provide the environment for AF generation, atrial size (which is more directly linked to AF) was not affected. Further mechanistic studies are required to investigate the role of *ASXL1*-CH in the development of AF, as presently there exists no murine models or genetic inferential studies examining this.

Given *JAK2* mutations are relatively rare within the CH population^62^, there has been insufficient evidence at the population level to reliably establish increased risk of AF; however, in a study conducted by Teng et al.^33^ in a cohort of 439 individuals with myeloproliferative neoplasms (including those with polycythaemia vera and essential thrombocytopaenia), who carry *JAK2*^*V617F*^ missense variants, 6.6% of patients developed AF over a median follow up of 6 years. We therefore highlight this as an area for future research, given that extrapolation of results from alternative populations cannot be reliably used to infer risk within the CH population, despite having a biologically plausible association.

Despite a lack of association of *DNMT3A*-CH with AF risk in studies conducted by Saadatagah et al.^32^ and Schuermans et al.^31^, there is evidence of an association both observationally and with genetic inferential studies. Ahn et al.^30^ reported a 45% increase in odds of AF with *DNMT3A*-CH (OR: 1.45, 95% CI: 1.09–1.93, *p* = 0.012), which is further corroborated by an MR study by Kar et al.^6^ (OR: 1.05, 95% CI: 1.02–1.07, *p* = 1.99 × 10^−4^), providing supportive evidence of causality. The mechanism for such an association is comparatively less well understood than the association observed with *TET2* mutations, however a further MR study has implicated larger right atrial size^65^ which may contribute to AF generation; in addition, Rauch et al.^51^ describe concordant macrophage phenotypes with loss of function mutations in *Tet2*^−/–^ and *Dnmt3a*^−/−^ mice, which suggests *DNMT3A-*CH and AF associations may also be driven by a pro-inflammatory phenotype, highlighting the need for future investigation in this area.

Associations between autosomal mCA, mLOX and mLOY with AF and flutter have been described within the UKB PheWAS conducted by Lin et al.^60^ (autosomal mCA: OR: 1.31, 95% CI: 1.17 − 1.46, *p* = 1.56 × 10^−6^; mLOX: OR: 1.66, 95% CI: 1.41−1.94, *p* = 3.70 × 10^−10^; mLOY: OR: 1.61, 95% CI: 1.51−1.71, *p* = 1.34 × 10^−51^). In a recent sensitivity analysis conducted by Lim et al.^71^, among 415,466 UKB participants, similar associations of mLOY with AF and flutter was observed among never smokers (HR: 1.07, 95% CI: 1.01–1.14, *p* = 0.029) and ever smokers (HR: 1.05, 95% CI: 1.01–1.11, *p* = 0.030) as well as men aged >60 and ≤60 years, however these were non-significant after Bonferroni correction. MR analysis in the same study provided further highly significant evidence of a causal relationship between mLOY and AF (HR: 1.15, 95% CI: 1.13–1.18, *p* = 1.52 × 10^−21^).

## Cardio-oncology

Cancer survivors have a heightened risk of CVD compared to the general population. A substantial proportion of this elevated cardiovascular risk has been attributed to the effects of cancer therapies on the cardiovascular system, high burden of shared risk factors and biological processes related to the cancer itself, such as systemic inflammation and prothrombotic states. Many of these factors also increase the likelihood of acquiring CH-associated driver mutations, including advancing age, tobacco smoking, chronic inflammation and the direct mutagenic effects of chemoradiation. In recent years, CH has become an area of increasing interest in cardio-oncology, as accumulating data suggest that cancer patients with CH mutations face higher risks of CVD and all-cause mortality, especially those carrying larger clones. This has prompted calls for evaluating CH as a potential biomarker to enhance cardiovascular risk stratification in cancer survivorship care^72,73^.

Recent studies indicate that patients with solid tumours display a higher prevalence of CH than the general population, with variations by cancer types^73–75^. For instance, in a large sequencing study of over 12,000 adults with non-haematological cancers, 23.1% of individuals over 80 years carried CH-related mutations detectable in cell-free DNA^74^. The most frequently mutated genes included *DNMT3A*, *TP53*, *TET2* and *PPM1D*, with gene patterns varying by tumour type; for example, *TP53* was the most frequent CH gene in melanoma and small-cell lung cancer, while *DNMT3A* mutations were most common in uterine and cervical cancers^74^. A separate analysis similarly reported that 25.1% of patients with non-haematological malignancies had at least one clonal somatic mutation^75^.

Cancer survivors commonly develop therapy-associated CH, a distinct form of clonal expansion characterised by mutations in DNA damage response (DDR) genes such as *ATM*, *CHEK1*, *CHEK2*, *TP53* and *PPM1D* that confer a selective growth advantage^2–4^. This form of CH is thought to be driven by the genotoxic stress induced by cytotoxic chemotherapy and radiotherapy, both of which promote the acquisition and expansion of somatic mutations. Anticancer therapies such as platinum agents, anthracyclines and thoracic radiation can promote the expansion of haematopoietic clones carrying driver mutations. In a large study of 24,146 cancer patients, Bolton et al.^2^ reported that external beam radiotherapy and regimens containing platinum and topoisomerase II inhibitors were most strongly correlated with CH in specific DDR pathway genes, including *TP53*, *PPM1D* and *CHEK2*. Consistent findings have been observed in other settings; for example, among patients with non-Hodgkin lymphoma undergoing autologous stem-cell transplantation and receiving anthracycline-based treatment, nearly 30% were found to have CH, with *PPM1D* and *TP53* representing the dominant mutations^76^.

Emerging evidence suggests that CH may also contribute to cardiotoxicity risk in cancer patients. In a small prospective study of 88 solid cancer patients treated with immune checkpoint inhibitors, CH more than doubled the risk of myocarditis (HR 2.74, 95% CI: 1.44–5.22, *p* = 0.002) and was a greater prognostic factor than dual therapy^77^. In a cohort of patients with breast cancer, sarcoma and multiple myeloma, CH was an independent predictor of doxorubicin-induced cardiotoxicity, even after accounting for conventional risk factors^78^. Supporting data from lymphoma cohorts show a similar pattern: *TET2*-mutant clones were independently associated with anthracycline-related cardiotoxicity^79^. Consistent findings have been reported in breast cancer patients treated with HER2-targeted therapies and chest radiotherapy, among whom CH, most commonly involving *DNMT3A* and *TET2* *mutations*, was detected in 38% of individuals and associated with an independent risk of therapy-related cardiomyopathy^72^. However, it remains uncertain whether these associations are driven by a selective expansion of clones carrying driver mutations following exposure to cytotoxic therapy^80,81^. An important next step in the field is understanding how genotoxic treatments shape clonal dynamics and influence cardiovascular outcomes.

Both CVD and CH are common in cancer patients, and CH is increasingly recognised as a contributor to cardiovascular vulnerability in this population. With growing evidence linking CH to adverse cardiac outcomes, it is emerging as a potentially useful biomarker for identifying cancer patients at heightened cardiovascular risk. Yet important uncertainties remain, including the mutation-specific and clone-size-dependent risks, the timing of clonal expansion and the degree to which therapy-associated CH differs biologically from age-related CH. Addressing these questions will be essential for defining whether and how CH should be incorporated into personalised cardio-oncology risk stratification and survivorship care.

## Research gaps and directions for future work

Existing literature evaluating the cardiovascular associations of CH has several key biases – reverse causation, confounding, survival bias and insufficient statistical power. We will discuss these issues and potential mitigations in the remainder of this section.

### Reverse causation

Studies reporting prevalence and cross-sectional associations of CH in cohorts selected on those illnesses (e.g. HF) are subject to reverse causation – it is not possible to distinguish the temporal relationship of CH with the CVD of interest (or its associated risk factors), which may in itself drive development of CH. Longitudinal analyses are essential to determine causal associations of CH with CVD.

### Residual confounding factors

There are many shared exposures that drive both CVD and CH, and insufficient adjustment for these variables will introduce confounding and may falsely augment observed relationships. In multivariate regression models with CVD as the outcome and CH as the main predictor, the usual confounding factors that are adjusted for are age, sex and smoking; however, some analyses have indicated that residual confounding by smoking may influence current estimates^20^. Furthermore, CH is associated with a wide variety of ageing-related traits and disorders, including CVD. It is possible that chronological age may not be representative of biological age or organ-specific age. Indeed, organ-specific ageing has been quantified using plasma protein signatures^82^, and it would be pertinent to assess biological age^46^ and organ-specific age as a confounding factor in the observed relationship between CH and CVD.

### Survival bias

The availability of large and deeply phenotyped longitudinal cohorts of unselected populations (e.g. UKB) allows mitigation of the aforementioned biases. However, these cohorts may be hampered by survival bias—in that individuals with the most unfavourable forms of CH may not survive to enter such cohorts, particularly those with recruitment in middle or older age (such as UKB).

### Statistical power attributed to rare mutations, small clones and phenotype definition

While research biobanks can provide large sample sizes, statistical power remains insufficient for rarer mutations. Furthermore, older technologies used at the time for sample analysis in these cohorts may miss small clones, which are increasingly recognised as of potentially prognostic importance. For example, Mutect2 is the main bioinformatics tool for CH detection, but current recommendations for this approach are the inclusion of mutations with ≥2% VAF, and for selected indels, such as *ASXL1* G646Wfs*12, ≥10% VAF^12^. In particular, *JAK2*, *SF3B1* and *U2AF1* hotspot mutations have lower coverage (median sequencing coverage of ≤31 reads) which limits the ability to detect clones with ≥2% VAF. ‘pileup’ is a more sensitive approach for detecting low-VAF mutations, particularly those supported by just 1–2 sequencing reads^83^, and it is able to identify mutations with ≥2% VAF missed by Mutect2^84^.

Phenotypic definitions amongst epidemiological studies, including genetic inferential studies, have been a source of controversy, particularly with regard to differences in the methodological techniques applied to ascertain CH in large-scale datasets. For example, a variety of approaches have been applied to classify CH within large datasets (such as whole genome sequencing^6^, whole exome sequencing^12^, targeted exome sequencing^9^ and mutational barcode-defined^20^ methods), yet there still remains a notable lack of an international consensus agreement on the most appropriate method of ‘calling’ CH, which has been identified to result in disease association underestimation^48^. We further argue that this may, at least in part, underlie the sometimes-conflicting genetic associations of CH with CVD outcomes. In addition, cardiovascular conditions are heterogeneous in terms of their aetiology and natural history; however, across most published reports, there is limited granularity of CVD definitions, which restricts inferences about biological mechanisms linking CH with disease-specific cardiovascular conditions.

### Establishing causality using Mendelian randomisation

Genetic inferential studies using MR can be used to reduce reverse causation and confounding biases and have a role for triangulation of evidence alongside other study types – however, methodological scrutiny of the assumptions of such analyses^85,86^ is warranted when drawing conclusions. In this setting, genetic variants associated with CH can be used as instrumental variables to generate causal estimates on downstream outcomes (e.g. CVDs). Validity of such genetic causal inference studies is highly sensitive to the reliability of the instrumental variables, particularly in the exposure genome-wide association studies (GWAS). It is also noteworthy that it is only feasible to assess overall-CH, *DNMT3A*-CH and mLOY as exposures in traditional MR (such as Inverse Variance Weighted MR). For these classes of CH, more than 20 genetic risk loci have been identified, and therefore, there is a sufficient number of genetic instruments to assess these CH classes as exposures in traditional MR. As for other classes of CH (*TET2*-CH, *ASXL1*-CH, *TP53*-CH, *JAK2*-CH, autosomal mCA and mLOX), less than 10 genetic risk loci have been identified, and therefore it is not feasible to assess these classes of CH as exposures using traditional MR techniques^6,9,10,41,65^. Finally, appropriate care must be taken to mitigate the effects of horizontal pleiotropy, that is, the instrumental variables affect outcomes (in this case, CVD) through pathways which are not via the exposure being examined (in this case, CH). For example, *SH2B3* mutations are known to promote *JAK2*-CH, but also in their own right have associations with CVD, hence, without appropriate adjustment, MR analyses may overcall associations of *JAK2*-CH with CVD^87^.

### Elucidating the mechanism of CH contributing to CVD development

Experimental models have been instrumental in furthering our understanding of pathophysiology in CH. Most studies have been based on bone marrow chimeric models involving lethal irradiation to empty the bone marrow; this then allows transplantation of a mix of wild-type and mutant bone marrow cells into the irradiated mouse in the proportion required to mimic the clonality of the condition. Irradiation is a destructive process which causes profound inflammation and can alter the physiology of the heart and great vessels. Clonal dynamics and expansion in the bone marrow are likely to be extremely different when competing within an empty bone marrow. Some studies transplant 100% mutant cells, which do not mimic clonality in CH^59^; as such, the action of mutant cells may be supra-pathophysiological or may miss the cell non-autologous effects or interactions with WT cells within the haematopoietic compartment or their progeny. Other studies focus on homozygous mutations, which rarely occur in CH (notably humans homozygous for *DNMT3A* mutations are virtually non-existent), and it is unclear how this gene-dosage effect relates to the mechanisms and consequences of heterozygotic mutations in humans.

### Association between novel classes of CH with CVD

An emerging class of CH without any identifiable driver gene mutations or chromosomal abnormalities has recently been described^20,47,88^. The association between this class of ‘driverless’ CH and CVD is not well understood. In a recent UKB study, individuals with CH based on the detection of passenger mutations demonstrated no association with CVD^20^. Similarly, a separate and recent study found no association between passenger mutation burden or clonal expansion with ischaemic stroke, coronary artery disease, or MI^89^. Nevertheless, these individuals with ‘barcoded’ CH encompass driver gene-mutant CH, mCAs and ‘driverless’ CH. Future work distinguishing ‘driverless’ CH as a distinct entity may help clarify our understanding of the traits and diseases, including CVD, associated with this class of CH. Furthermore, current research has focused upon myeloid CH; however, CH driven by mutations in genes associated with lymphoid malignancies has been found to cause immune dysregulation^90^ and to be associated with autoimmune disease, yet associations with CVDs of lymphoid CH are currently understudied.

### Concluding remarks

Moving forward, studies should focus on large, unselected populations with longitudinally detected disease-specific cardiovascular outcomes. Multicohort analyses are needed to allow sufficient statistical power to study rarer CH driver mutations. Modern high-resolution sequencing technologies may provide new insights into the clinical significance of small clones. While there is a role for MR analyses, these should be interpreted in conjunction with other study types and scrutinised with regard to relevant biases and instrument strengths. A broader consensus on the definition of CH would support universal cross-comparability of both observational and genetic studies. Pre-clinical models remain critical to advancing biological understanding of the cardiovascular consequences of CH and to identifying potential druggable targets.

## Clinical implications

At present, there are no widespread CH population screening programmes or any guidelines that advocate for its testing. Despite this, there do exist clinics which demonstrate the feasibility of screening and managing the sequelae of a CH diagnosis^91^. These clinics could provide a cohort of patients who could directly contribute to further research efforts by including them in biorepositories^92^; this would be particularly useful given that genomic sequencing is not commonplace in most healthcare systems and therefore identification of individuals with CH for research is limited. With growing recognition of the consequences of CH, as well as clinical trials which will guide how to treat it on the horizon, this raises the potential of CH screening programmes.

### Treatment: preventing sequelae or halting progression

**Fig. 2:**
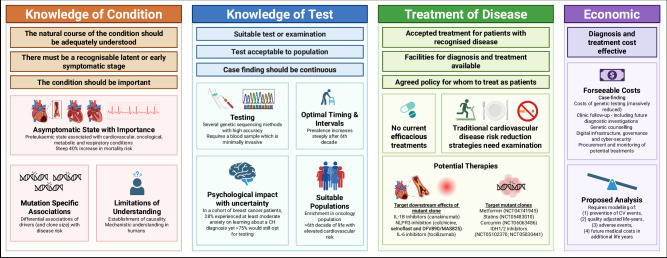
CH screening: considerations and current status within the Wilson & Jungner framework. CH clonal haematopoiesis, CV cardiovascular. Figure was created in BioRender. Morley, A. (2026) https://biorender.com/3gi3e5f

In 1968, Wilson and Jungner proposed a set of criteria for assessment of the suitability of screening for any given condition, incorporating factors related to the condition being screened, the screening test and economic implications^93^, as summarised in Fig. 2. For screening to be justifiable, the availability of efficacious treatments with clear guidance on whom to treat is integral. As there are currently no treatments proven by clinical trials to halt CH or its consequences, this is an important knowledge gap. However, mounting pre-clinical evidence implicates augmented inflammation, and particularly the NLRP3 inflammasome, in the pathogenesis of CH-driven CVD, which may pave the way for therapeutic strategies. In animal models of *TET2*-CH, the worsening of atherosclerosis^52^, HF^66^ and AF^69^ were all halted by inhibition of the NLRP3 inflammasome, which produces IL-1β. Furthermore, in a post-hoc analysis of the CANTOS^94^ trial, patients with *TET2*-CH derived greater benefit from neutralisation of IL-1β with canakinumab, supporting the notion of this therapeutic approach. This has motivated the IMPACT^95^ trial, which is recruiting patients with clonal cytopenia of unknown significance to assess the effect of canakinumab on cardiovascular outcomes alongside haematological outcomes such as progression to myeloid cancer, mutational burden and blood counts (NCT05641831). Although promising, the cost and fatal infections associated with canakinumab may be a barrier to its implementation, and other methods of targeting inflammation may be necessary. Colchicine is thought to reduce cardiovascular events by similar mechanisms, and notably, colchicine reduced atherogenesis in animal models of *TET2*-CH and colchicine users in the UKB and MGBB had lower rates of MI^23^. Additionally, the effect of alternative NLRP3 inhibitors, including selnoflast (ISRCTN10520571) and DFV890/MAS825 (NCT06097663) are being studied in patients with CH and coronary artery disease. Lastly, IL-1β drives IL-6 production, with the latter thought to be causal in CVD. IL-6 inhibition in animal models^96^, and genetic perturbations in humans^97,98^ both reduce atherosclerotic disease. As there are IL-6 inhibitors, such as tocilizumab, already in clinical use, this is another promising option for future clinical trials. An overview of ongoing clinical trials is shown in Table 3.

**Table 3:** Ongoing clinical trials in CH / CCUS.

Trial	Intervention type	Drug/therapy	Status	Dates	Clinical trial code
CHIP-HF: colchicine in heart failure patients with CHIP	Small molecule	Colchicine (microtubule inhibitor)	Ongoing	Aug 2021–(ongoing)	EudraCT 2021-001508-13
A study of enasidenib in people with clonal cytopenia of undetermined significance	Small molecule	Enasidenib (IDH2 inhibitor)	Active, not recruiting	Oct 2021–Oct 2026 (est)	NCT05102370
Repurposing metformin as a leukaemia-preventive drug in CCUS and LR-MDS	Small molecule	Metformin (biguanide)	Recruiting	Dec 2021–Feb 2026 (est)	NCT04741945
Ivosidenib for patients with clonal cytopenia of undetermined significance and mutations in IDH1	Small molecule	Ivosidenib (IDH1 inhibitor)	Active, not recruiting	Apr 2022–Jan 2030 (est)	NCT05030441
A study to assess the safety, processing by the body, and effects on the body following 4 weeks of selnoflast dosing in participants with coronary artery disease	Small molecule	Selnoflast (NLRP3 inhibitor)	Completed	Jul 2022–May 2023	ISRCTN10520571
Canakinumab for the prevention of progression to cancer in patients with clonal cytopenias of unknown significance, IMPACT study	Biologic (monoclonal antibody)	Canakinumab (IL-1β inhibitor)	Recruiting	Feb 2023–Dec 2028 (est)	NCT05641831
DFV890 and MAS825 for inflammation reduction in CHD patients with CHIP	Small molecule & biologic (monoclonal antibody)	DFV890 (NLRP3 inhibitor) & MAS825 (anti–IL-1β monoclonal antibody)	Recruiting	Feb 2024–(ongoing)	NCT06097663
Statins in patients with clonal cytopenia of undetermined significance (CCUS) and myelodysplastic syndromes (MDS)	Small molecule	Atorvastatin/rosuvastatin (HMG-CoA-reductase inhibitors)	Recruiting	Feb 2024–May 2027 (est)	NCT05483010
Curcumin to improve inflammation and symptoms in patients with clonal cytopenia of undetermined significance, low risk myelodysplastic syndrome, and myeloproliferative neoplasms	Supplement	Curcumin (Supplement; anti-inflammatory/anti-oxidant)	Recruiting	Mar 2024 – Mar 2027 (est)	NCT06063486
TECTONIC: IL-1β Inhibition in TET2-CHIP and ASCVD	Biologic (monoclonal antibody)	Canakinumab (IL-1β inhibitor)	Not yet recruiting	Jul 2025–Apr 2030 (est)	NCT06691217

*ASCVD* atherosclerotic cardiovascular disease, *CCUS* clonal cytopenia of undetermined significance, *CH* clonal haematopoiesis, *CHD* coronary heart disease, *CHIP* clonal haematopoiesis of indeterminate potential, *LR-MDS* low-risk myelodysplastic syndromes.

Screening could also be of value if treatments are discovered which halt progression of CH to myeloid malignancies, or even expansion in VAF – with larger clone sizes a key factor in determining leukaemic^99–101^ and cardiovascular^18^ risk. Numerous clinical trials are underway in this regard. Strategies being tested include metformin (NCT04741945) and vitamin C, both of which are associated with a lower risk of CH in observational studies^11,102,103^, and attenuate clonal expansion in animal models^11,104,105^. Other trials are assessing the potential of statins (NCT05483010) or curcumin (NCT06063486) in reducing progression of clonal cytopenias or low-risk myelodysplasia through suppression of inflammation. Lastly, specific mutations are being targeted, with studies of IDH1/2 inhibitors with known efficacy in IDH1/2 mutant AML being trialled in the context of IDH1/2 mutant CH (NCT05102370 and NCT05030441). A multi-faceted approach to discovering efficacious, safe treatments to prevent both the cardiovascular and haematological consequences of CH is therefore underway.

### Identifying CH in the clinic

CH can be identified through several genetic sequencing methods with high accuracy^106^, which only require a blood sample, and so is minimally invasive to the patient. Sequencing will become more accessible as costs continue to decline, and low-cost assays for CH detection have been developed^107^. Notably, this assay has not been validated for clinical use, which should be a priority when low-cost solutions are developed to enable benefits to patients. Perhaps more of a barrier to consider is the psychological impact of receiving a CH diagnosis, and with it the uncertain risk of developing multi-system diseases with high morbidity and mortality. This was emphasised in a study of over 500 breast cancer patients surveyed on their opinions of CH testing^108^. Twenty-eight percent of participants experienced at least moderate anxiety on learning about CH, with the associated diseases and lack of treatments being factors that influenced this. Despite this, the majority of patients (>75%) reported that they would opt for testing for themselves and that a CH diagnosis would prompt them to change their behaviours and seek medical attention to reduce their health risks. Overall, this study suggests that within certain populations, such as oncology, CH screening may be viewed as acceptable. Given the enrichment of CH after cancer treatments^109–112^, this may be a suitable high-risk population for screening in the future. However, the perspectives of patients with recent breast cancer diagnosis may not be representative of the wider population, and acceptability would need to be assessed in other high-risk populations being considered for screening. Further research will guide who this would benefit, but could include those at high risk for CVD, with premature CVD or with a family history of leukaemia or CH.

## Conclusion

CH is emerging as an important risk factor for CVDs, and more broadly for overall mortality risk; however, such risk is heterogeneous and often mutation and clone-size-specific. Our understanding of this nuanced relationship remains in its infancy, but as we look to transition from bench-to-bedside, there are knowledge gaps and inconsistencies which must be reconciled. In order to facilitate future efforts in examining associations of CH genetically with outcomes (including CVD), we highlight the need for an international consensus on methods of defining CH with respect to driver genes, mutation types and filtering strategies. CVD associations of mutation subtypes must then be clarified in longitudinal cohorts with sufficient statistical power. There is a potential role for screening and risk-stratification at a population level, which may become a reality with ongoing clinical trials designed to combat CVD risk in CH.

## Data Availability

No datasets were generated or analysed during the current study.
